# Lomatogonium Rotatum for Treatment of Acute Liver Injury in Mice: A Metabolomics Study

**DOI:** 10.3390/metabo9100227

**Published:** 2019-10-14

**Authors:** Renhao Chen, Qi Wang, Lanjun Zhao, Shilin Yang, Zhifeng Li, Yulin Feng, Jiaqing Chen, Choon Nam Ong, Hui Zhang

**Affiliations:** 1 National Pharmaceutical Engineering Center for Solid Preparation in Chinese Herb Medicine, Jiangxi University of Traditional Chinese Medicine, Nanchang 330002, China; chen98@live.com (R.C.); zhaolanjun1991@163.com (L.Z.); yangshilin9705@hotmail.com (S.Y.); 2 State Key Laboratory of Innovative Drug and Efficient Energy-Saving Pharmaceutical Equipment, Nanchang 330006, China; wangqi19760615@hotmail.com (Q.W.); fengyulin2003@hotmail.com (Y.F.); 3 NUS Graduate School for Integrative Sciences and Engineering, National University of Singapore, Singapore 119077, Singapore; jiaqing_chen@u.nus.edu; 4 Saw Swee Hock School of Public Health, National University of Singapore, Singapore 117549, Singapore; ephocn@nus.edu.sg; 5 NUS Environmental Research Institute, National University of Singapore, Singapore 117411, Singapore

**Keywords:** *Lomatogonium rotatum*, metabolomics, acute liver injury, LC-MS, GC-MS

## Abstract

*Lomatogonium rotatum (L.)* Fries ex Nym (LR) is used as a traditional Mongolian medicine to treat liver and bile diseases. This study aimed to investigate the hepatoprotective effect of LR on mice with CCl_4_-induced acute liver injury through conventional assays and metabolomics analysis. This study consisted of male mice (*n* = 23) in four groups (i.e., control, model, positive control, and LR). The extract of whole plant of LR was used to treat mice in the LR group. Biochemical and histological assays (i.e., serum levels of alanine transaminase (ALT) and aspartate transaminase (AST), and histological changes of liver tissue) were used to evaluate LR efficacy, and metabolomics analysis based on GC-MS and LC-MS was conducted to reveal metabolic changes. The conventional analysis and metabolomic profiles both suggested that LR treatment could protect mice against CCl_4_-induced acute liver injury. The affected metabolic pathways included linoleic acid metabolism, α-linolenic acid metabolism, arachidonic acid metabolism, CoA biosynthesis, glycerophospholipid metabolism, the TCA cycle, and purine metabolism. This study identified eight metabolites, including phosphopantothenic acid, succinic acid, AMP, choline, glycerol 3-phosphate, linoleic acid, arachidonic acid, and DHA, as potential biomarkers for evaluating hepatoprotective effect of LR. This metabolomics study may shed light on possible mechanisms of hepatoprotective effect of LR.

## 1. Introduction

Natural medicines have been continuously studied and utilized to treat various diseases, including liver diseases [1,2,3]. The dried whole herb of *Lomatogonium rotatum (L.)* Fries ex Nym (LR) is one key component of Digeda, which includes more than 20 species of Mongolian medicinal plants and is commonly used as the main or auxiliary medicine in many prescriptions [4]. One well-known natural habitat of LR is the central and eastern Inner Mongolia, which has a long history of utilizing LR for treatment of diseases [5,6,7]. Being capable of clearing heat, eliminating dampness, and detoxification, LR is efficient in the treatment of various symptoms, such as hepatobiliary fever, headache, jaundice, and other diseases [6,7]. Previous studies have identified the bioactive compounds of LR, including xanthones, flavonoids, iridoids, glycosides, etc. [8,9]. A recent study revealed that flavonoids extracted from LR may be beneficial for reducing blood lipid levels and preventing obesity [10]. Our previous study suggested that 50% ethanol fraction of LR, which mainly contained xanthones and flavonoids, might have promising hepatoprotective effect [9]. Though LR has been used for clinical treatment of hepatobiliary diseases, there is still a lack of solid support from animal experimental studies, which are designed for better understanding of the mechanisms. There is an urgent need to start addressing this knowledge gap with the relevant emerging technologies, such as metabolomics.

By revealing profiles and changes of metabolites in various organisms, metabolomics has been applied to reveal pathways, identify biomarkers, and illustrate mechanisms of various effects from endogenous and exogenous sources [11,12]. Metabolomics is strongly recommended as a promising technology to study the evidence-based traditional Chinese medicine (TCM), which has been gaining attentions because of its beneficial applications in many health-related areas, such as complementary therapy, and early intervention in disease and drug discovery [13,14,15,16]. Recent studies have demonstrated the capabilities of applying metabolomics to reveal possible mechanisms behind effects of herbal medicine and processed TCM. For example, the hepatoprotective effect of Celosiae Semen extract [17], the antifatigue effect of Danggui Buxue Tang [18], and the renal protective effect of genipin derived from the fruit of *Gardenia jasminoides* [19], have been investigated through metabolomics by using rats or mice models. The findings (e.g., changes of metabolites and pathways) could help advance the knowledge of these effects and may promote better applications of these medicines in the future. 

This study aimed to investigate the hepatoprotective effect of LR on mice with CCl_4_-induced acute liver injury through comprehensive evaluation of biochemical and histological assays, metabolic changes, and metabolic pathway analysis. Gas chromatography and liquid chromatography coupled with time-of-flight mass spectrometer (GC-TOF MS and LC-TOF MS) were used to provide comprehensive coverage of metabolites in untargeted metabolomics analysis.

## 2. Results and Discussion

### 2.1. Efficacy of LR Treatment

In this study, CCl_4_ was used to induce acute liver injury in mice. As a typical liver poison, CCl_4_ has been widely used to induce liver injury models for studies of hepatoprotective drugs as well as liver necrosis and cirrhosis. The C–Cl bond of CCl_4_ can be destroyed by the cytochrome P450 enzymes in hepatocytes to produce trichloromethyl free radicals, which further leads to the generation of more toxic free radicals (e.g., superoxide anions and hydroxide anions) through peroxide chain reaction [20,21]. These free radicals are responsible for degeneration and necrosis of liver cells [22].

**Figure 1:**
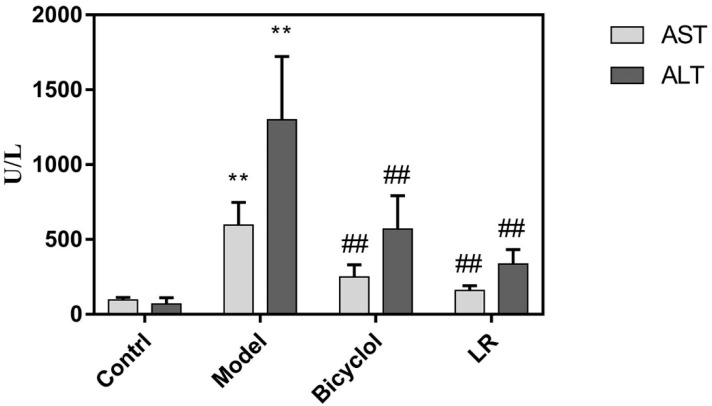
The serum levels of ALT and AST in mice from control, model, bicyclol, and LR groups (Mean ± SD). **: *p* < 0.01 (in comparison with control group, *t*-test); ^##^: *p* < 0.01 (in comparison with model group, *t*-test).

Serum levels of alanine transaminase (ALT) and aspartate transaminase (AST) as well as histology of liver tissue were used to evaluate efficacy of LR treatment on mice with acute liver injury. The samples were collected from the four groups of mice: control, model, positive control, and LR. Transaminase can be released into blood when liver cells are injured by CCl_4_, of which process the typical indicators are elevated levels of ALT and AST in serum [23]. As shown in Figure 1, the serum levels of AST and ALT were both significantly higher in the model group than the control group, indicating that the model was successfully established. In comparison with the model group, the serum levels of AST and ALT were both significantly lower in the positive control group and the LR group (Figure 1). This might suggest that LR showed beneficial effects on liver functions as the well-known medicine bicyclol with respect to the indicators of AST and ALT.

**Figure 2:**
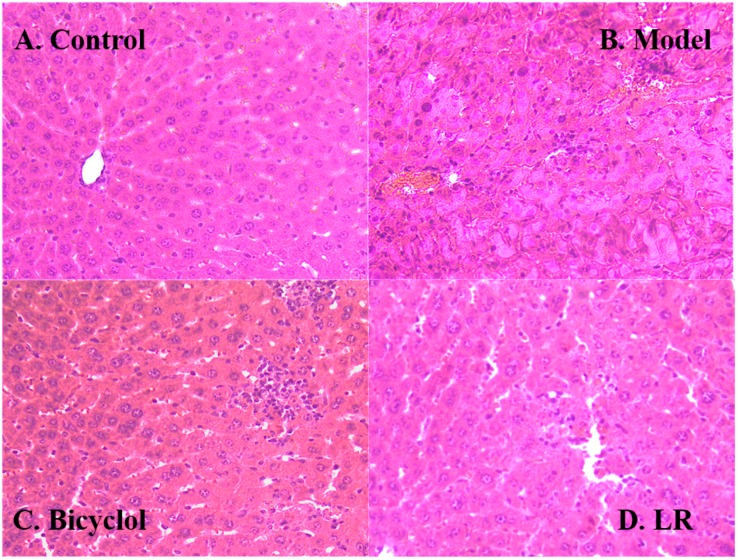
Representative hematoxylin and eosin (HE) stained sections (200×) of the liver of mice from the groups of control (**A**), model (**B**), bicycle (**C**), and LR (**D**).

The histology of liver tissue showed obvious differences between the control group and the model group (Figure 2A,B). In the control group, liver lobule structure was neat and liver cells were well-arranged, indicating that the mice were in good condition, although hepatic sinus was slightly expanded. In the model group, liver tissue showed clear signs of a series of injuries, such as liver lobular necrosis, disordered arrangement of hepatic plate, and liver nucleus shrinkage. These further confirmed that the model was successfully established. In comparison with the control group and the model group, liver tissue in the positive control group and the LR group both presented signs of relief from liver injuries (Figure 2C,D). Furthermore, the liver tissue showed a very similar hepatic lobule between the LR group and the control group. However, LR could not fully recover liver functions from the adverse effects of CCl_4_, given that slight necrosis and inflammatory cell infiltration could still be observed in the LR group.

Biochemical and histological assays both showed that bicyclol protected mice against liver injury, which was consistent with a previous study [24]. Our finding highlighted that LR had comparable effects as bicyclol with regards to the serum levels of AST and ALT, as well as histologic changes of liver tissue. These preliminary findings suggested that LR might have the capability to protect liver against acute chemical injury, but more studies are needed to explore this kind of application of LR.

### 2.2. Metabolic Responses of Mice to LR Treatment

#### 2.2.1. Data Quality and Identification of Metabolites

**Figure 3:**
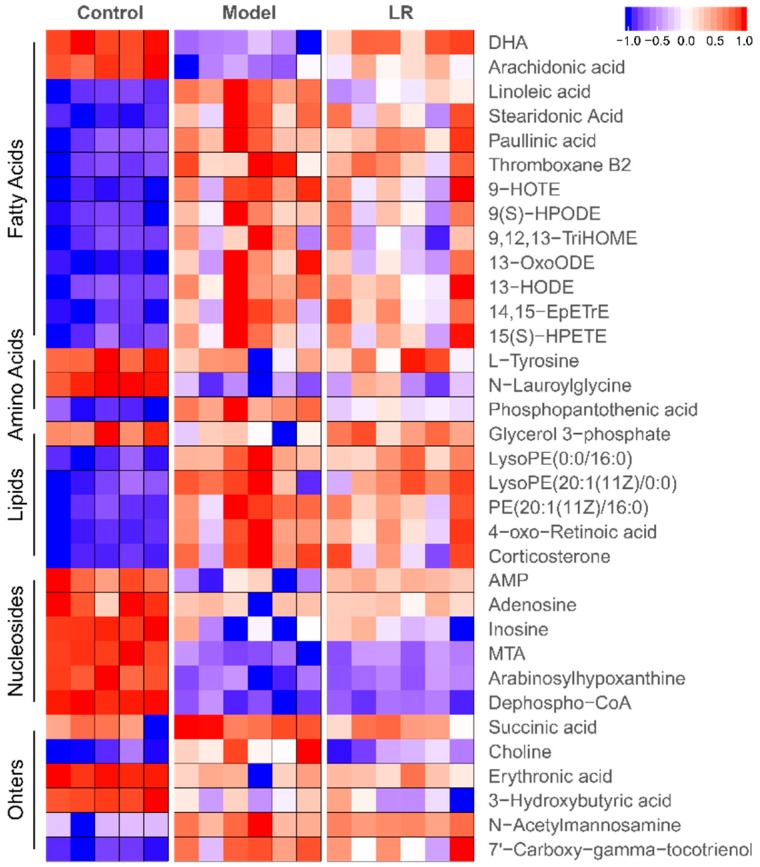
Heatmap of metabolites in the liver of mice from the control, model, and LR groups. Data (peak areas) were normalized between –1 and 1 (blue—the lowest level; red—the highest level). DHA: Docosahexaenoic acid; AMP: Adenosine monophosphate; MTA: 5’-Methylthioadenosine.

The data quality was evaluated through differences of major peaks in quality control (QC) samples and distribution of QC samples in principal component analysis (PCA) plot [25,26]. The typical total ion chromatograms (TICs) of QC samples in GC-MS and LC-MS are shown in Supplementary Figure S1. The low relative standard deviations (RSDs) of peak areas in GC-MS (2.0%–6.6%) and LC-MS (< 16.4%), as well as the low RSDs of retention times in GC-MS (0.01%–0.06%) and LC-MS (< 1.0%), both indicated high reproducibility of sample analysis (Tables S1 and S2). After data screening, the eligible features were analyzed through PCA, in which the clustered QC samples further confirmed the high data quality (Figure S2). In plots of PCA and orthogonal projections to latent structures discrimination analysis (OPLS-DA), the well separated three groups (i.e., control, model, and LR groups) indicated distinct profiles of features in these groups (Figure S2). The crucial features contributing to the separation of these groups were selected according to variable importance in projection (VIP) values (> 1, OPLS-DA) and *p*-values (< 0.05, *t*-test). The corresponding metabolites (*n* = 34) of these features were identified as fatty acids, amino acids, lipids, nucleosides and others (Figure 3 and Figure S3, Table S3). 

#### 2.2.2. Changes of Metabolites

The levels of metabolites in the control, model, and LR groups are shown in heatmap (Figure 3). It was found that 13 metabolites, including two fatty acids (DHA and arachidonic acid), two amino acids (l-tyrosine and N-lauroylglycine), six nucleosides, glycerol 3-phosphate, 3-hydroxybutyric acid, and erythronic acid, had higher levels in the control group than the model and LR groups, while these metabolites generally had relatively higher levels in the LR group than the model group. The other metabolites, including most of fatty acids and lipids, phosphopantothenic acid, N-acetylmannosamine, succinic acid, 7’-carboxy-gamma-tocotrienol, and choline, had lower levels in the control group than the model and LR groups, while these metabolites generally had relatively lower levels in the LR group than the model group. These data revealed that the levels of metabolites in the LR group were generally between the ones in the control group and the model group, highlighting that LR treatment might help to eliminate the adverse effects of CCl_4_ by regulating the levels of these metabolites. The data of metabolic changes suggested that LR might have hepatoprotective effect on mice with CCl_4_-induced acute liver injury, which was consistent with the findings from biochemical and histological assays.

**Figure 4:**
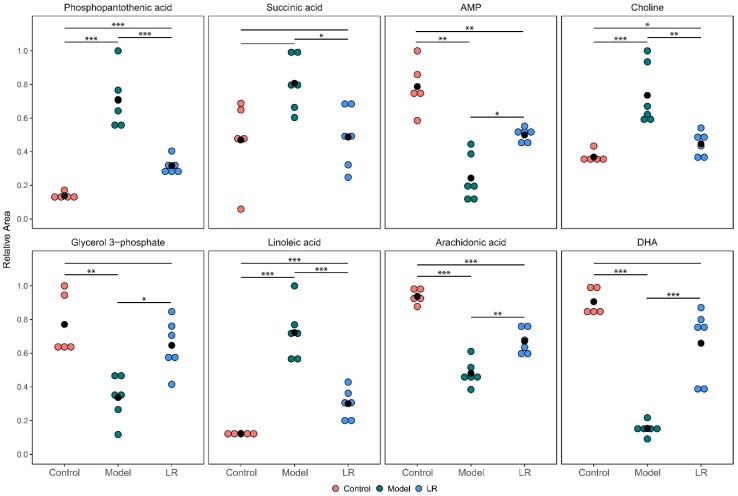
Relative levels of the selected eight metabolites in the liver of mice from the control, model, and LR groups. The hypothesis test for the difference between two means was conducted through *t*-test. *: *p* < 0.05; **: *p* < 0.01; ***: *p* < 0.001.

The metabolites with significantly different levels (*p* < 0.05) between the model group and the LR group were selected out of the 34 metabolites to further elucidate the effect of LR treatment. The selected eight metabolites included phosphopantothenic acid, DHA, arachidonic acid, linoleic acid, glycerol 3-phosphate, adenosine monophosphate (AMP), succinic acid, and choline (Figure 4). The differences of the levels of these metabolites between the control group and the LR group were much smaller than the ones between the control group and the model group. Moreover, there were no statistically significant differences between the control group and the LR group with regards to the levels of three metabolites, which were DHA, glycerol 3-phosphate, and succinic acid. These eight metabolites might be used as potential biomarkers to evaluate LR effects on mice with CCl_4_-induced liver injury.

### 2.3. Metabolic Pathways and Biological Functions of Metabolites

**Figure 5:**
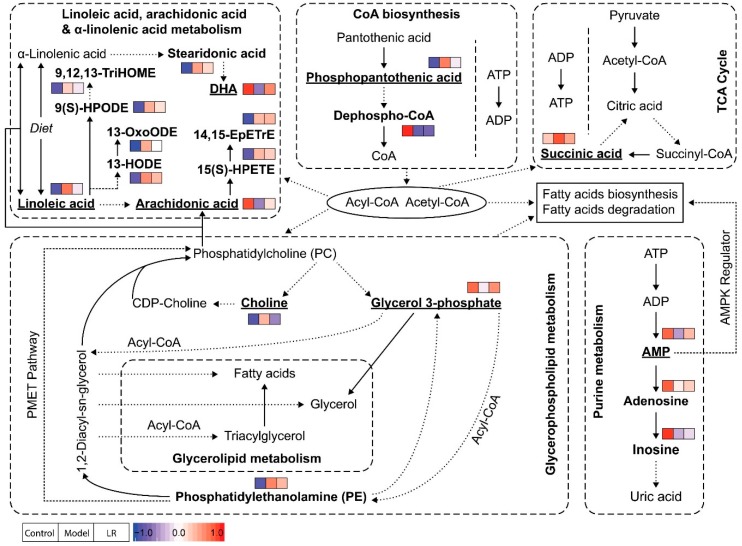
Possible pathways related to the affected metabolites in the liver of mice from the groups of control, model, and LR. The metabolites identified in this study were highlighted in bold, and the eight key metabolites were underlined. Relative levels of identified metabolites were normalized and presented in color (blue—the lowest level; red—the highest level) following the order of control group, model group, and LR group.

The pathway analysis with the 36 metabolites identified six major metabolic pathways, including linoleic acid metabolism, arachidonic acid metabolism, pantothenate and CoA biosynthesis, glycerophospholipid metabolism, tricarboxylic acid cycle (TCA cycle), and purine metabolism (Figure S4). The details of metabolic pathways in terms of the identified metabolites are presented in Figure 5.

Phosphopantothenic acid is an intermediate in the coenzyme A (CoA) biosynthesis pathway which requires five steps with the utilization of pantothenic acid, cysteine, ATP, and various enzymes [27]. Previous studies showed that pantothenic acid (vitamin B5), the precursor of phosphopantothenic acid, had higher levels in serum of mice with ethanol-induced liver injury [28], and it could protect liver from oxidative stress caused injury, including valproic acid-induced hepatotoxicity [29,30]. Pantothenic acid was not identified in our study, but its metabolite (phosphopantothenic acid) had significantly higher levels in the model group than the control group, while dephospho-CoA, the precursor of CoA, had significantly lower levels in the model group. In CoA biosynthesis, the first step (pantothenic acid to phosphopantothenic acid) and the fourth step (transfer of AMP moiety of ATP to form dephospho-CoA) are the two rate-limiting reactions [27]. Our data suggested that the CoA biosynthesis in mice of the model group was inhibited, possibly because of the lack of ATP. Accordingly, CoA might have lower levels in the model group, but this was not confirmed in the untargeted metabolomics analysis. CoA is an essential cofactor in living organisms and it participates in various metabolic pathways as acyl or acetyl group carrier [27,31]. The lack of CoA can inhibit the processes requiring acyl or acetyl group, such as fatty acid metabolism and the TCA cycle.

Succinic acid is a central metabolite in the TCA cycle which produces reduced electron carriers (NADH and FADH2) and ATP. In our study, succinic acid had significantly higher levels in the model group than the control group, which was consistent with a previous study showing elevated levels of succinic acid in liver of mice exposed to CCl_4_ [32]. The increased levels of succinic acid might indicate that the TCA cycle was disturbed and hence less ATP was produced. Considering the roles of CoA in the TCA cycle and ATP in CoA biosynthesis, the inhibition of these two processes in mice of the model group could be highly related. Besides its traditional role in TCA cycle, the function of succinic acid as a signaling molecule in various diseases, including liver damage, has been reported [33]. With G-protein coupled receptor 91 (GPR91) as the receptor, succinic acid regulates many cell functions, for example, increased levels of succinic acid can lead to development of liver fibrosis through activation of hepatic stellate cells [33,34,35]. 

AMP is a nucleotide and can be interconverted to the other two nucleotides (i.e., adenosine diphosphate (ADP) and adenosine triphosphate (ATP)). These nucleotides have multiple essential roles in metabolism, for example, they are subunits of nucleic acid in polynucleotides (e.g., DNA and RNA) synthesis and principal energy carriers in energy metabolism (e.g., the TCA cycle). When ATP is consumed to produce AMP, as reflected by high AMP-to-ATP ratio, AMP-activated protein kinase (AMPK) can be activated to maintain cellular energy homeostasis by promoting processes that generate ATP (e.g., fatty acid oxidation) and inhibiting processes that consume ATP (e.g., fatty acid synthesis) [36,37]. An earlier study reported increased AMPK activity in liver of rats with CCl_4_-induced liver injury within 24 h and suggested that AMPK could stimulate autophagy to maintain cellular homeostasis [38]. However, another study revealed that AMPK was primarily activated at the liver regeneration stage in mice with CCl_4_-induced liver injury, but not in early stage of liver injury [39]. In our study, AMP and two of its metabolites (e.g., adenosine and inosine) had lower levels in the model group than the control group. Compared with the control group, the lower levels of AMP and the higher levels of most fatty acids in the model group suggested that AMPK might not be activated yet in our study. Furthermore, the low levels of AMP together with the high levels of succinic acid could indicate poor energy state for mice in the model group, as supported by the study reporting decreased ATP levels and lower ATP-to-ADP ratio in liver of CCl_4_-induced cirrhotic rats [40]. The comprehensive study of AMP, ADP, ATP, TCA cycle, and fatty acid metabolism via targeted analysis together with AMPK at different stages of liver injury may provide better understanding of liver injury and regeneration. 

In glycerophospholipid metabolism, choline and glycerol 3-phosphate have multiple roles in biosynthesis and degradation of phosphatidylcholine (PC), a major component of the cell membrane. In the liver, PC is responsible for assembly and secretion of very low density lipoproteins (VLDLs) that constitute of triglycerides, cholesterol, and phospholipids, and impaired PC biosynthesis (i.e., low PC level) induces liver damage because of a reduction in circulation of VLDLs [41,42]. PC is synthesized from choline via CDP-choline pathway and from phosphatidylethanolamine (PE) via PE N-methyltransferase (PEMT) pathway, with the CDP-choline pathway dominating the production [41]. Choline is only required in the CDP-choline pathway, while glycerol 3-phosphate and PE can participate in the two pathways. PC hydrolysis by a series of phospholipases can generate choline, glycerol 3-phosphate, and fatty acids. Our study revealed significantly higher levels of choline and significantly lower levels of glycerol 3-phosphate in the model group than the control group. The increased levels of choline and CDP-choline in liver of mice with CCl_4_-induced liver injury were reported in NMR-based metabolomics studies, but one of the studies reported increased levels of choline/PC [43,44]. The higher levels of choline might be due to the inhibition of PC biosynthesis through the CDP-choline pathway and/or hydrolysis of PC, which also led to lower levels of PC. The step from CDP-choline to PC might be inhibited in PC biosynthesis in mice of the model group, which is supported by previous studies showing higher levels of choline and CDP-choline as well as increased expression of choline kinase in the mice/rat models with drug or CCl_4_-induced liver injury [43,44,45]. The possible hydrolysis of PC in mice of the model group is supported by the activation of phospholipases in mice models with drug or CCl_4_-induced liver injury [45,46]. Glycerol 3-phosphate is a key precursor for PC and PE biosynthesis in glycerophospholipid metabolism and can be hydrolyzed to generate glycerol in glycerolipid metabolism. The consumption of glycerol 3-phosphate in these processes could be a possible reason for its lower levels in the model group than the control group. Our data and pathway analysis suggested that PC might have lower levels and PE might have higher levels in the model group than the control group, though only limited PE were identified in our study. The increased levels of PC and the decreased levels of PE in the liver of mice with drug-induced liver injury have been reported, and the study also suggested that accumulated PE could be due to the inhibition of the PEMT pathway, as evidenced by the down-regulation of PEMT in liver [45].

Linoleic acid, arachidonic acid, and DHA are three essential fatty acids actively involved in polyunsaturated fatty acids (PUFAs) biosynthesis, in which linoleic acid and α-linolenic acid are metabolized to arachidonic acid and DHA, respectively [47,48]. It is known that omega-6 PUFAs derived from linoleic acid and omega-3 PUFAs derived from α-linolenic acid can influence development of fatty liver, hepatic steatosis, and hepatic microcirculation [47]. It is also known that both arachidonic acid and DHA have anti-inflammatory effects against liver injury or liver disease [49,50,51,52]. In our study, linoleic acid had significantly lower levels in the model group than the control group, while its metabolite (arachidonic acid) showed significantly higher levels in the model group than the control group. The distinct comparison indicated the possible inhibition of linoleic acid metabolism, which could be attributed to the deactivation of Δ-6 desaturases and elongases in oxidative microenvironment. The oxylipins originated from linoleic acid (e.g., 9(S)-HPODE, 13-HODE) and arachidonic acid (e.g., 15(S)-HPETE) all had elevated levels in the model group, which might suggest the existence of lipid peroxidation via CCl_4_-induced free radicals [53,54,55]. In comparison with the control group, the lower levels of DHA together with the higher levels of stearidonic acid in the model group might be associated with the inhibition of α-linolenic acid metabolism.

## 3. Materials and Methods

### 3.1. Materials

Whole plant of LR was collected from the Inner Mongolia of China. ALT and AST kits were purchased from Nanjing Jiancheng Bioengineering Institute (Nanjing, China). Acetonitrile and methanol (LC-MS grade) were purchased from Fisher Scientific (Shanghai, China). Bicyclol tablets and formic acid (analytical grade) were purchased from Daxing Biomedicine Industrial Park (Beijing, China) and China Chemical Reagent Co., Ltd. (Shanghai, China), respectively. Carboxymethylcellulose sodium salt (CMC-Na) and carbon tetrachloride (CCl_4_) were purchased from Xilong Scientific Co., Ltd. (Shantou, China). N-methyl-N-(trimethylsilyl)-trifluoroacetamide (MSTFA), methoxyamine hydrochloride, ammonium acetate, luteolin, n-alkanes, and pyridine were obtained from Sigma-Aldrich (Shanghai, China). HP-20 macroporous resin was purchased from Mitsubishi Chemical Corporation (Tokyo, Japan). Deionized water was produced by the Milli-Q water system (Bedford, MA, USA).

### 3.2. Preparation of LR Extract

Fresh LR herb was washed, dried, and then crushed into powder. LR powder (500 g) was extracted twice with 70% ethanol (5 L) for 2 h each time. The extract (10 L) was concentrated to 500 mL under vacuum at 60 °C after centrifugation at 5000 rpm for 10 min, the supernatant of the extract was loaded onto HP-20 macroporous resin, which was then washed with 50% ethanol (2 L). The eluate was concentrated under vacuum at 60 °C and then freeze-dried. Carboxymethylcellulose sodium salt (CMC-Na) solution was used to dissolve the LR extract. In this study, the LR suspension was administered to mice at 200 mg/kg according to clinical dosage.

### 3.3. Study Design

The Institute of Cancer Research (ICR) mice (male, *n* = 23, body weight 18–22 g) from Shrek Jingda Laboratory Animal Company (Hunan, China) were randomly divided into 4 groups after a week of adaptation. The 4 groups included a control group (*n* = 5), a model group (*n* = 6), a positive control group (*n* = 6), and a LR group (*n* = 6). The same living conditions, such as constant room temperature (25 ± 1 °C), humidity (40%–50%), and controlled light/dark cycle (12/12 h), were maintained for the mice in the 4 groups. Through oral administration, mice in the LR group were fed with LR extract (200 mg/kg), and mice in the positive control group were fed with bicyclol (20 mg/kg), which is known as an effective drug for treatment of liver injury [24]. In comparison, mice in the control group and the model group were fed with saline through oral administration. The treatment was conducted for 7 days. After that, CCl_4_ solution (0.1 mL/100 g) dissolved in corn oil was administered to mice in the model, positive control and LR groups through intraperitoneal injection, while corn oil was administered to mice in the control group. After 12 h, the blood and liver samples of mice in the 4 groups were collected.

### 3.4. Sample Preparation

All animal experiments were conducted in accordance with the guidelines of the Review Committee of Animal Care and Use at Jiangxi University of Traditional Chinese Medicine (JZLLSC2019_0078).

The mice were anesthetized with isoflurane before sample collection. Blood samples were taken from eye sockets of mice and transferred to Eppendorf tube with no additives to stand for 1 h. After that, blood samples were centrifuged at 4000 rpm for 10 min and the serum (supernatant) samples were collected. The serum samples were stored at −80 °C prior to ALT and AST analysis. 

The mice were decapitated immediately after blood collection. The liver samples of mice were taken and washed with ice saline. After removing excess saline with filter paper, the liver was evenly divided into two sections. The section for histological assay was stored in 10% formalin prior to analysis. The section for metabolomics analysis was weighed and then stored at −80 °C prior to analysis. 

### 3.5. Biochemical and Histological Assays

The serum levels of ALT and AST in mice were determined by automatic biochemical analyzer (Hitachi 7100, Tokyo, Japan) following the instruction. The histopathological images of the liver tissues of mice were recorded at 200× magnification under microscope with digital camera (Olympus Microscope CX31 and DP 12 Micro-Systems Digital Imaging, Olympus, Japan). For histological assay, the liver tissue was embedded in paraffin after rinsing off formalin with Milli-Q water. The paraffin section with liver tissue was subjected to hematoxylin and eosin (H&E) staining and then histological assay.

### 3.6. Metabolomics Analysis

#### 3.6.1. Sample Pretreatment

Metabolomics analysis was applied for mice in the control, model, and LR groups. The method for sample pretreatment was adopted from previous studies [26,56]. In brief, liver tissue samples were freeze-dried and weighed before extraction. A mixture of methanol and water (4:1, *v*/*v*; 250 µL) containing luteolin (10 µg/mL) as internal standard was used to extract metabolites. Stainless steel beads were added into extraction vials to enhance efficiency. The sample was homogenized (25 Hz) at 4 °C for 10 min and then sonicated in ice water for 10 min. After extraction, the sample was centrifuged (14,000 rpm) at 4 °C for 10 min, and then the supernatant was divided into two fractions for LC-MS and GC-MS analysis, respectively. For LC-MS analysis, 100 µL supernatant was used without further treatment. Prior to GC-MS analysis, 10 µL supernatant was dried and derivatized. The derivation was conducted with methoxyamine in pyridine (5 mg/L) for 2 h at 60 °C and MSTFA for 16 h at 37 °C.

#### 3.6.2. LC-MS and GC-MS Analysis

A Shimadzu UHPLC and an AB Sciex quadrupole time-of-flight mass spectrometer (TripleTOF^®^ 5600, Framingham, MA, USA) were used for LC-MS analysis. The separation of metabolites was performed on an ACQUITY UPLC C18 column (100 × 2.1 mm, 1.7 µm, Waters, Milford, MA, USA) at 40 °C with flow rate of 0.3 mL/min. Milli-Q water with 0.1% formic acid (A) and acetonitrile (B) were used as mobile phases for both positive and negative electrospray ionization (ESI) modes. The gradient of mobile phase B was as follow, 2%–30% (0–3 min), 30%–60% (3–5 min), 60%–80% (5%–15 min), 80%–100% (15–16 min), 100% (16–19 min), 100%–2% (19–20 min) and 2% (20–25 min). The mass range was from 50 to 1250 *m*/*z* for both full scan (TOF MS) and MS/MS scan, which were conducted simultaneously. The most intensive 8 ions from each full scan were selected as precursor ions for MS/MS scans at collision energy (CE) of 40 eV with collision energy spread (CES) of 15 eV.

An Agilent 7890A GC with a 7200 quadrupole time-of-flight mass spectrometer was used for GC-MS analysis. Separation was performed on a HP-5 MS UI column (30 m × 0.25 × 0.25 mm, Agilent, Santa Clara, CA, USA) with helium as carrier gas (1 mL/min). Temperatures of the inlet and transfer line were 250 and 280 °C, respectively. The oven temperature program was as follow: 90 °C held for 1 min, 90–130 °C (20 °C /min), 130–280 °C (6 °C /min), 280–300 °C (25 °C /min), and 300 °C held for 6 min. The solvent cut time was 4.5 min. Electron ionization (EI) ion source was used and its temperature was maintained at 230 °C. The mass range of TOF MS scan was from 50 to 800 *m*/*z*.

Several approaches were adopted from previous studies as quality assurance/quality control (QA/QC) [25,26]. TOF MS was calibrated to maintain high mass accuracy when every 5 samples were analyzed. QC samples (*n* = 5), which were prepared by pooling and aliquoting samples, were analyzed together with liver tissue samples in LC-MS and GC-MS analysis. One QC sample was injected for analysis when 5 samples were analyzed. QC samples were also analyzed before and after the batch analysis of samples. Relative standard deviations (RSDs) of retention times and intensities of 12 typical peaks in QC samples, as well as distribution of QC data in PCA were used to evaluate data quality.

#### 3.6.3. Data Analysis

The data analysis was adopted from previous studies [26,57]. In brief, XCMS and MZmine were used to process LC-MS and GC-MS data, respectively. The abundances of aligned features were normalized through the internal standard (luteolin) and sample weight. The features were subjected to further statistical analysis only when they had 100% detection frequencies (DFs) together with low RSDs (<30%) in at least one group. The missing values were replaced with half minimum of abundances of features. PCA and OPLS-DA were performed in SIMCA-P. Student’s *t*-test was performed in SPSS 20.0. 

#### 3.6.4. Metabolites Identification and Pathway Analysis

The features playing key roles in differentiating study groups were selected when their scores of VIP in OPLS-DA model were above 1 and their *p*-values in *t*-test were lower than 0.05 [26,58]. Human Metabolome Database (HMDB) and NIST library were used for identification of metabolites corresponding to the selected features in LC-MS and GC-MS analysis, respectively. The metabolic pathways associated with the identified metabolites were established through the integration of Kyoto Encyclopedia of Genes and Genomes (KEGG), Small Molecule Pathway Database (SMPDB), and MetaboAnalyst 3.0 [59,60,61].

## 4. Conclusions

This study investigated hepatoprotective effect of LR on mice with CCl_4_-induced acute liver injury through conventional assays together with GC-MS and LC-MS metabolomics. The results of biochemical and histological assays as well as metabolic changes both demonstrated a potential hepatoprotective effect of LR. The metabolomics analysis revealed that LR treatment could help restore the disturbed metabolic pathways, such as linoleic acid metabolism and glycerophospholipid metabolism. Our study suggested eight metabolites as potential biomarkers and could shed light on the mechanisms of the hepatoprotective effect of LR.

## Supplementary Materials

The following are available online at https://www.mdpi.com/2218-1989/9/10/227/s1, Figure S1: Total ion chromatograms of QC samples and selected top 12 peaks, A: GC-MS, B: LC-MS (ESI+), C: LC-MS (ESI–). Figure S2: Plots of PCA and OPLS-DA. A: PCA plot of GC-MS data (R^2^X: 0.774; Q^2^: 0.603); B: OPLS-DA plot of GC-MS data (R^2^X: 0.717; R^2^Y: 0.948; Q^2^: 0.631); C: PCA plot of LC-MS data (R^2^X: 0.62; Q^2^: 0.53); D: OPLS-DA plot of LC-MS data (R^2^X: 0.737; R^2^Y: 0.878; Q^2^: 0.815). Figure S3: MS/MS spectra (ESI+ and ESI–) of identified metabolites and the comparison with major fragments of metabolites in HMDB database. Figure S4: Result of metabolic pathway analysis through MetPA software. Table S1: The peak areas and retention times of top 12 peaks in QC samples (GC-MS). Table S2: The peak areas and retention times of top 12 peaks in QC samples (LC-MS). Table S3: Identified metabolites and their relative levels in the control, model, and LR groups.
