# Supplementary material for: Lomatogonium Rotatum for Treatment of Acute Liver Injury in Mice: A Metabolomics Study
Source: Metabolites. 2019 Oct 14;9(10):227. doi: 10.3390/metabo9100227 (PMC6836280; doi:10.3390/metabo9100227)
Supplement: Supplementary file 1 [file metabolites-09-00227-s001.pdf]

Lomatogonium Rotatum for Treatment of Acute Liver Injury in Mice: A Metabolomics Study

Renhao Chen [1], Qi Wang [2], Lanjun Zhao [1], Shinlin Yang [1], Zhifeng Li [1,*], Yulin Feng [2], Jiaqing Chen [3], Choon Nam Ong [4] and Hui Zhang [5,*]

[1] National Pharmaceutical Engineering Center for Solid Preparation in Chinese Herb Medicine, Jiangxi University of Traditional Chinese Medicine, Nanchang 330002, China
[2] State Key Laboratory of Innovative Drug and Efficient Energy-Saving Pharmaceutical Equipment, Nanchang 330006, China
[3] NUS Graduate School for Integrative Sciences and Engineering, National University of Singapore, 119077, Singapore
[4] Saw Swee Hock School of Public Health, National University of Singapore, 117549, Singapore
[5] NUS Environmental Research Institute, National University of Singapore, 117411, Singapore
* Correspondence: Hui Zhang, zhanghui@u.nus.edu; Zhifeng Li, lizhifeng1976@hotmail.com

Figure Captions:

Figure S1. Total ion chromatograms of QC samples and selected top 12 peaks, A: GC-MS, B: LC-MS (ESI+), C: LC-MS (ESI-).

Figure S2. Plots of PCA and OPLS-DA. A: PCA plot of GC-MS data (R^2X: 0.774; Q^2: 0.603); B: OPLS-DA plot of GC-MS data (R^2X: 0.717; R^2Y: 0.948; Q^2: 0.631); C: PCA plot of LC-MS data (R^2X: 0.62; Q^2: 0.53); D: OPLS-DA plot of LC-MS data (R^2X: 0.737; R^2Y: 0.878; Q^2: 0.815).

Figure S3. MS/MS spectra (ESI+ & ESI-) of identified metabolites and the comparison with major fragments of metabolites in HMDB database.

Figure S4. Result of metabolic pathway analysis through MetPA software.

Table Captions:

Table S1. The peak areas and retention times of top 12 peaks in QC samples (GC-MS).

Table S2. The peak areas and retention times of top 12 peaks in QC samples (LC-MS).

Table S3. Identified metabolites and their relative levels in the control, model and LR groups.

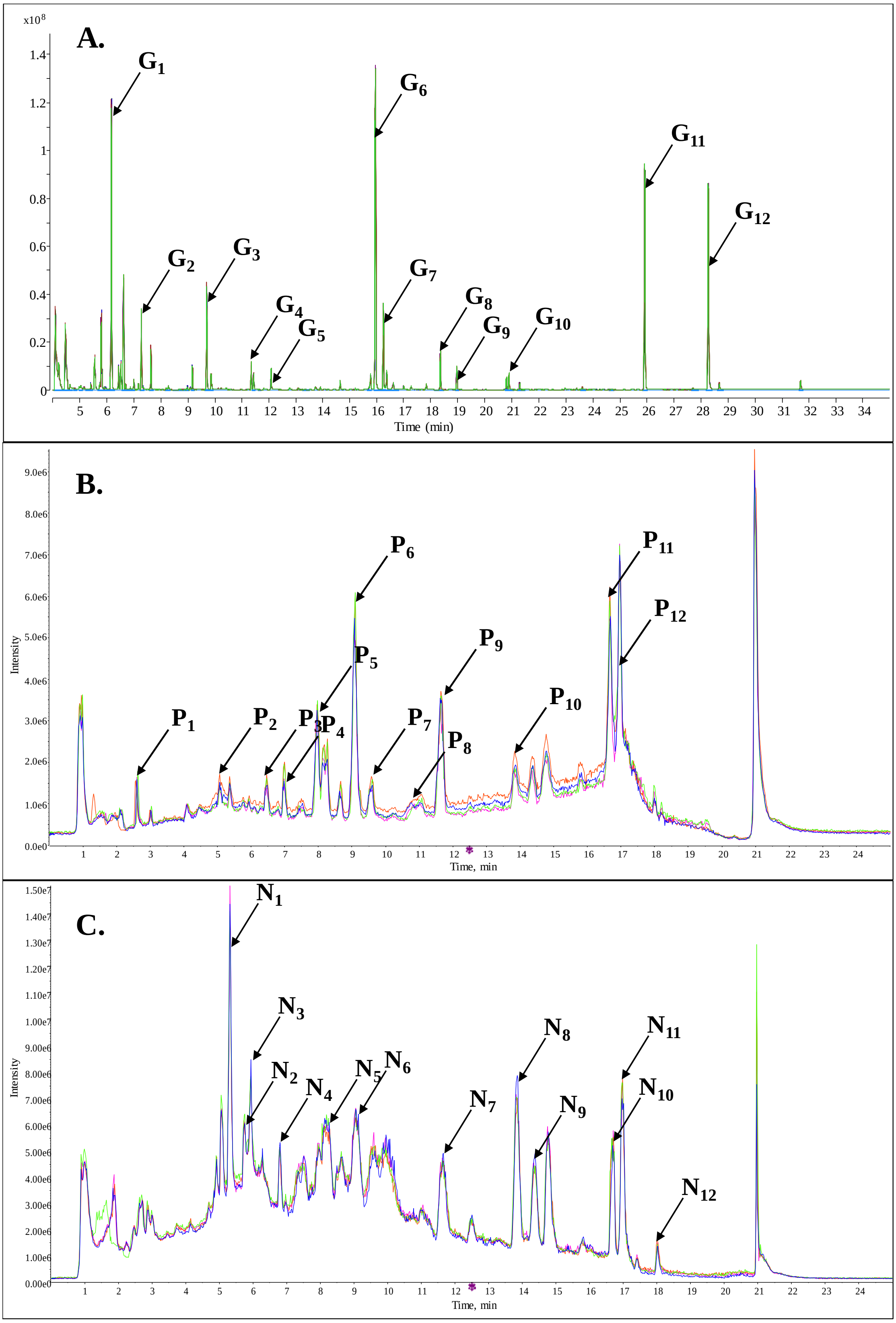

Figure S1. Total ion chromatograms of QC samples and selected top 12 peaks, A: GC-MS, B: LC-MS (ESI+), C: LC-MS (ESI-).

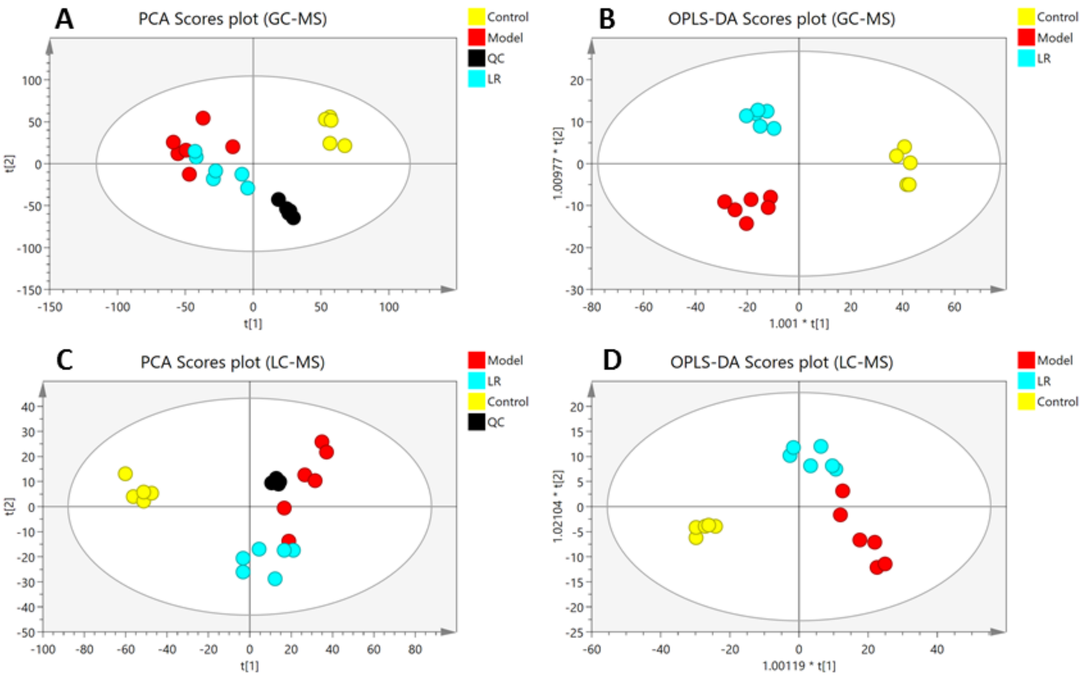

Figure S2. Plots of PCA and OPLS-DA. A: PCA plot of GC-MS data (R^2X: 0.774; Q^2: 0.603); B: OPLS-DA plot of GC-MS data (R^2X: 0.717; R^2Y: 0.948; Q^2: 0.631); C: PCA plot of LC-MS data (R^2X: 0.62; Q^2: 0.53); D: OPLS-DA plot of LC-MS data (R^2X: 0.737; R^2Y: 0.878; Q^2: 0.815).

MS/MS data from HMDB

DHA

59.0133	67.0547	81.0704	83.0861	93.0704	107.0861
109.1017	121.1017	127.0759	133.1017	135.1174	147.1174
161.1330	163.1487	187.1487	201.1643	203.1800	241.1956
255.2113	283.2426	309.2218	327.2324		

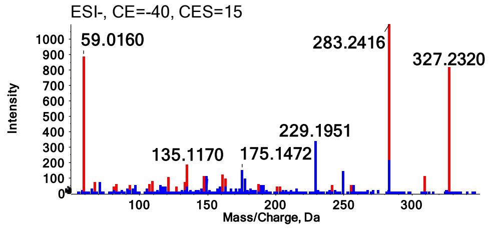

MS/MS data from HMDB

Arachidonic acid

59.0133	83.0861	177.1643	205.1956	231.2112	259.2425
285.2218	303.2324				

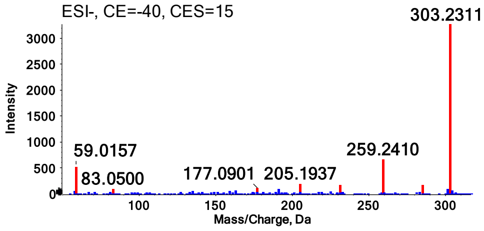

MS/MS data from HMDB

Linoleic acid

59.0133	71.0861	243.2113	261.2218	279.2324

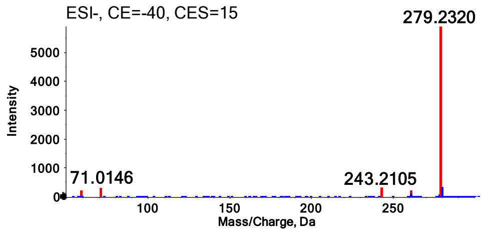

MS/MS data from HMDB

Stearidonic Acid

67.0548	79.0548	81.0704	121.1017	135.1174	151.0759

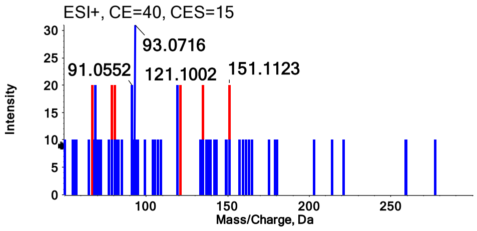

MS/MS data from HMDB

Paullinic acid

291.2688	309.2794

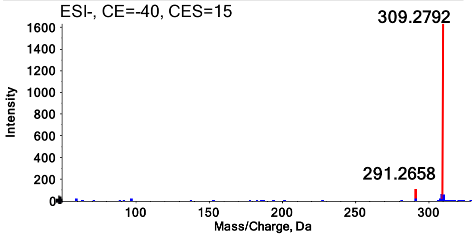

MS/MS data from HMDB

Thromboxane B2

71.0861	99.0810	251.2011	333.2066	351.2171	369.2277

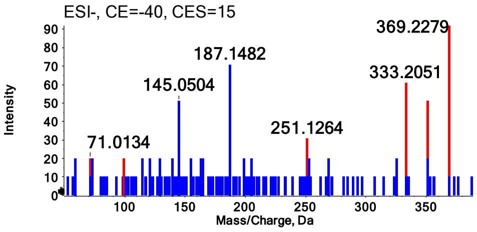

MS/MS data from HMDB

9-HOTE

67.0542	79.0542	81.0699	93.0699	121.1012	179.1430

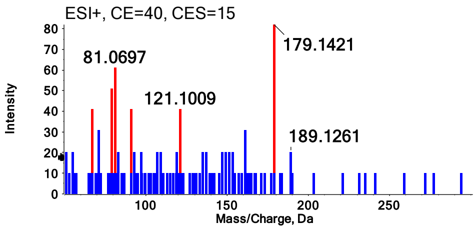

MS/MS data from HMDB

9(S)-HPODE

125.0972	135.1179	167.1078	249.2224	293.2122

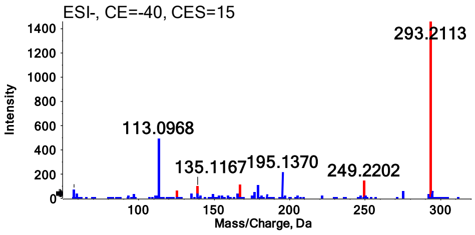

MS/MS data from HMDB

9,12,13-TriHOME

99.0815	127.0765	183.1390	209.1183	211.1340	229.1445
293.2122	311.2228	329.2333			

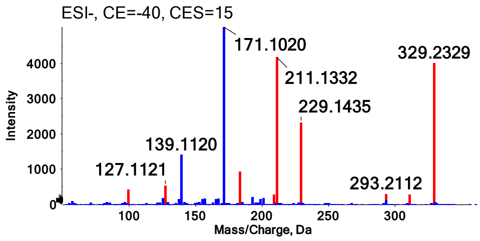

MS/MS data from HMDB

13-OxoODE

55.0542	67.0542	83.0855	121.0648	135.1168	151.1117
161.0961	179.1430				

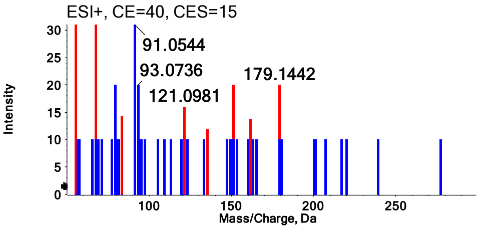

Figure S3. MS/MS spectra (ESI+ & ESI-) of identified metabolites and the comparison with major fragments of metabolites in HMDB database.

MS/MS data from HMDB

13-HODE

59.0133	113.0966	177.1279	179.14359	181.1592	195.1385
233.2269	259.2062	277.2168	295.2273		

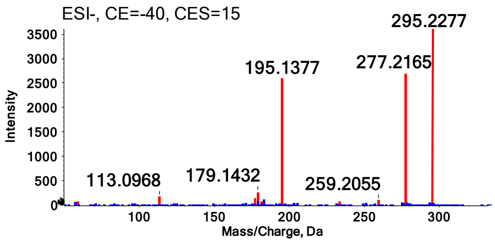

MS/MS data from HMDB

15(S)-HPETE

59.0139	71.0866	203.1441	273.2224	317.2122

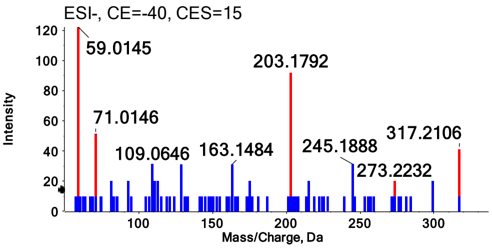

MS/MS data from HMDB

Phosphopantothenic acid

78.9585	96.9691	209.0215	280.0586	298.0692

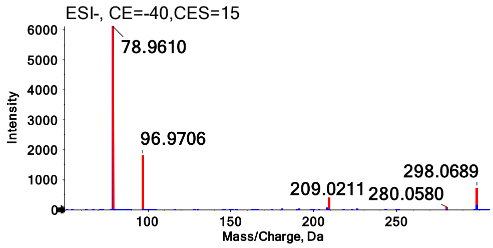

MS/MS data from HMDB

LysoPE(20:1(11Z)/0:0)

139.0155	155.0104	367.3207	447.2870	508.3398

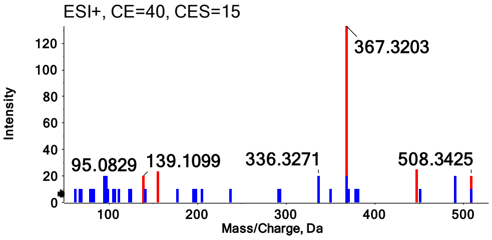

MS/MS data from HMDB

4-oxo-Retinoic acid

119.0861	145.0653	199.1123	201.1279

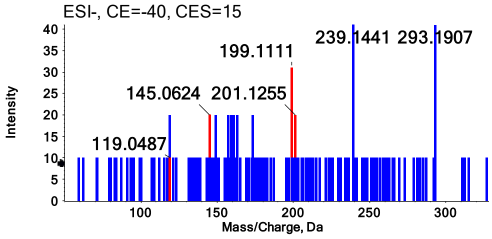

MS/MS data from HMDB

14, 15-EpETrE

55.0542	67.0542	79.0542	81.0699	93.0699	107.0855
119.0855	121.1011	123.0804	133.1011	137.0961	147.1168
149.1324	161.1325	175.1481	189.1274	201.1274	219.1379
285.2213	303.2319				

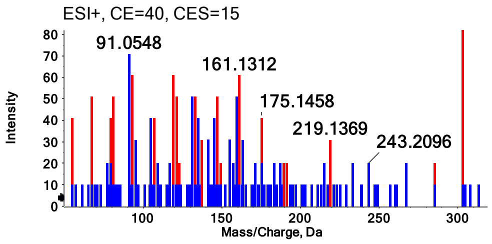

MS/MS data from HMDB

N-Lauroylglycine

74.0248	212.2020	256.1919

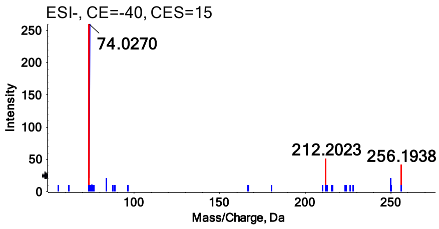

MS/MS data from HMDB

LysoPE(0:0/16:0)

57.0335	71.0855	85.1012	155.1794	198.0526	216.0632
239.2369	257.2475	313.2737	393.2401	436.2822	454.2928

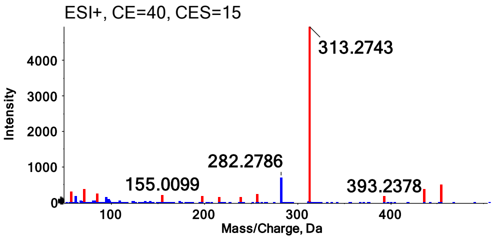

MS/MS data from HMDB

PE(20:1(11Z)/16:0)

313.2742	746.5700

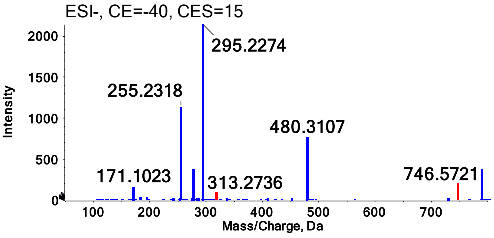

MS/MS data from HMDB

Corticosterone

59.0133	269.1905	285.1854

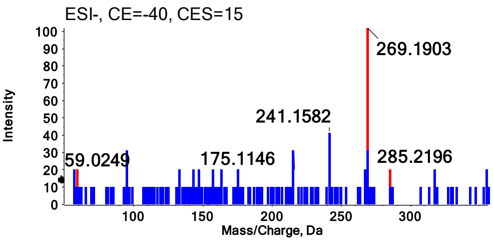

Figure S3. Continued.

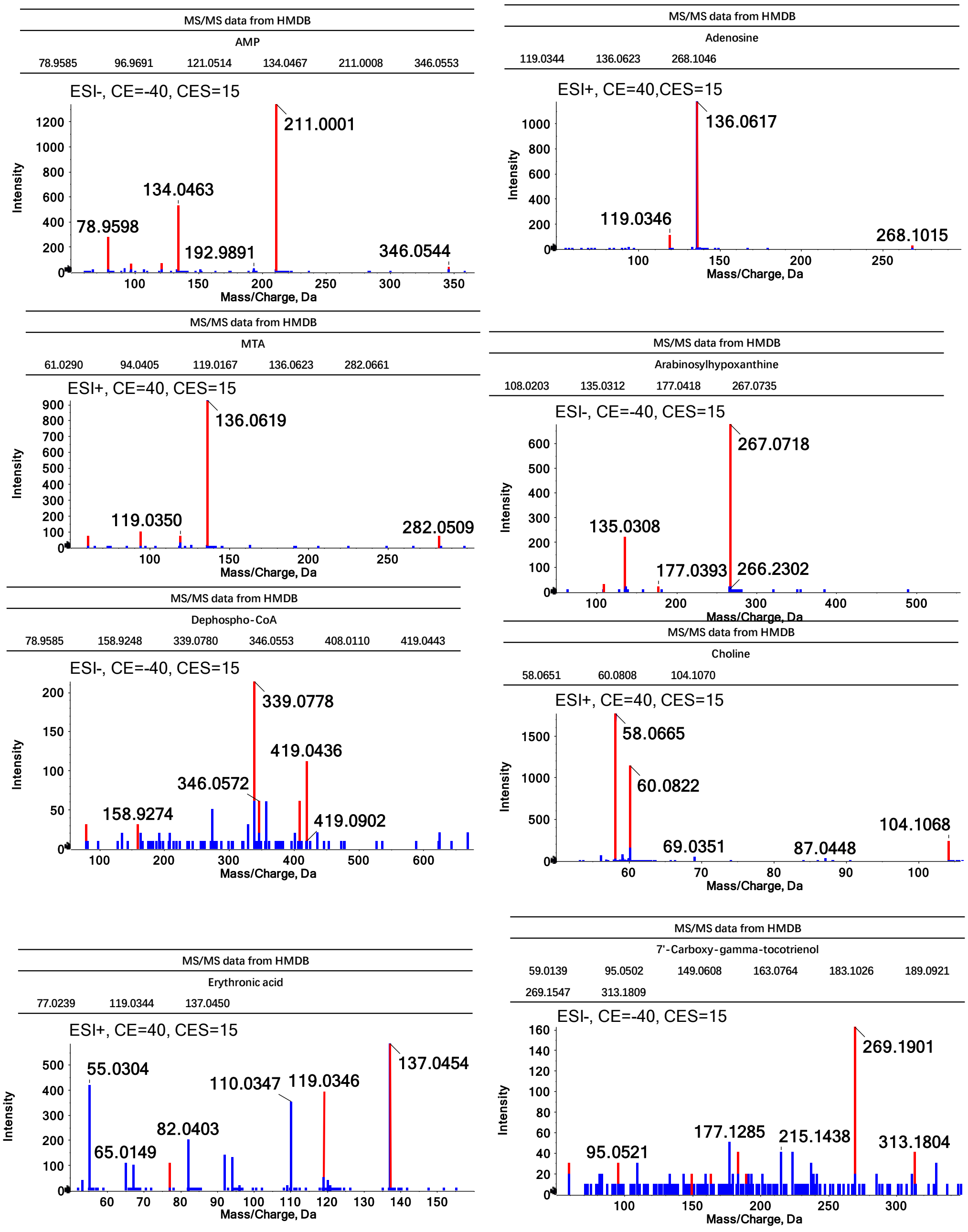

Figure S3. Continued.

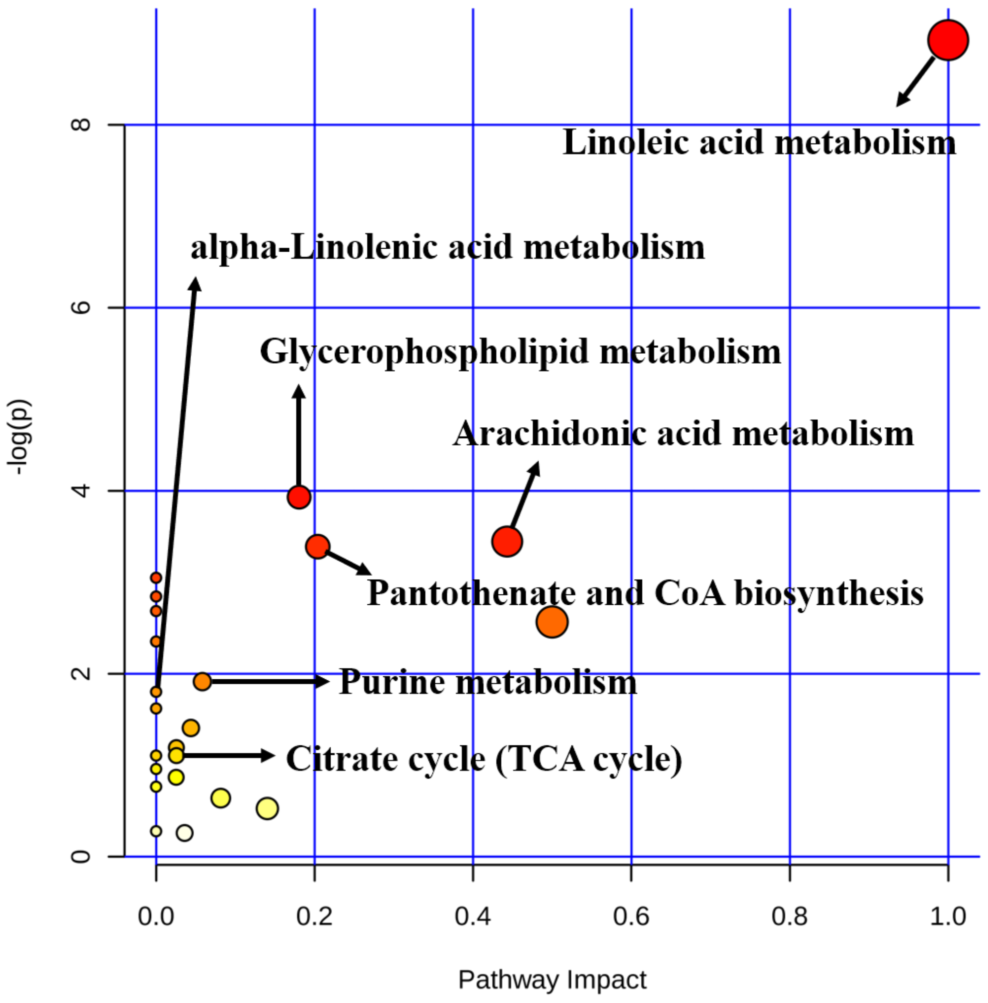

Figure S4. Result of metabolic pathway analysis through MetPA software.

Table S1. The peak areas and retention times of top 12 peaks in QC samples (GC-MS).

NO.	Peak area		Retention time	
	Mean ± SD (× 10^7)	RSD%	Mean ± SD	RSD%
G1	26.75 ± 0.69	2.60	6.17 ± 0.00	0.06
G2	5.04 ± 0.17	3.29	7.29 ± 0.00	0.04
G3	10.08 ± 0.27	2.68	9.70 ± 0.00	0.03
G4	1.86 ± 0.11	5.94	11.35 ± 0.00	0.02
G5	1.49 ± 0.10	6.55	12.10 ± 0.00	0.02
G6	37.69 ± 0.31	0.83	15.95 ± 0.00	0.03
G7	7.59 ± 0.15	1.97	16.25 ± 0.00	0.02
G8	3.01 ± 0.07	2.47	18.35 ± 0.00	0.02
G9	2.08 ± 0.07	3.29	18.97 ± 0.00	0.02
G10	1.34 ± 0.07	4.97	20.89 ± 0.00	0.01
G11	23.73 ± 0.49	2.08	25.93 ± 0.00	0.01
G12	22.88 ± 0.46	2.00	28.28 ± 0.00	0.01

Table S2. The peak areas and retention times of top 12 peaks in QC samples (LC-MS).

NO.	LC-MS [a]	M/Z	Intensity		Retention time	
			Mean ± SD (× 10^4)	RSD%	Mean ± SD	RSD%
P1	ESI+	166.09	43.10 ± 3.50	8.12	2.60 ± 0.03	0.96
P2	ESI+	287.06	26.11 ± 2.03	7.76	5.07 ± 0.00	0.10
P3	ESI+	274.27	46.72 ± 4.18	8.94	6.44 ± 0.00	0.08
P4	ESI+	432.24	28.85 ± 3.35	11.63	6.98 ± 0.01	0.14
P5	ESI+	520.34	62.24 ± 9.44	15.17	7.98 ± 0.01	0.10
P6	ESI+	496.34	229.53 ± 18.02	7.85	9.09 ± 0.01	0.06
P7	ESI+	522.35	29.46 ± 3.94	13.36	9.60 ± 0.01	0.12
P8	ESI+	601.33	7.14 ± 0.62	8.72	10.83 ± 0.05	0.47
P9	ESI+	524.37	117.52 ± 6.16	5.24	11.67 ± 0.01	0.08
P10	ESI+	329.25	17.12 ± 2.80	16.35	13.85 ± 0.01	0.04
P11	ESI+	760.58	56.53 ± 5.12	9.06	16.68 ± 0.01	0.03
P12	ESI+	758.57	111.25 ± 11.75	10.56	16.96 ± 0.01	0.03
N1	ESI-	285.04	113.50 ± 6.33	5.58	5.07 ± 0.00	0
N2	ESI-	514.28	561.74 ± 39.28	6.99	5.32 ± 0.01	0.09
N3	ESI-	453.29	102.40 ± 3.97	3.87	5.93 ± 0.01	0.08
N4	ESI-	471.06	61.13 ± 3.88	6.36	6.79 ± 0.00	0
N5	ESI-	564.33	93.70 ± 5.80	6.19	8.28 ± 0.01	0.06
N6	ESI-	540.33	155.90 ± 11.07	7.10	9.11 ± 0.01	0.06
N7	ESI-	480.31	143.84 ± 4.98	3.46	11.59 ± 0.01	0.05
N8	ESI-	327.23	282.50 ± 20.99	7.43	13.84 ± 0.01	0.04
N9	ESI-	303.23	159.25 ± 11.68	7.33	14.37 ± 0.01	0.04
N10	ESI-	255.23	69.30 ± 8.03	11.58	16.68 ± 0.01	0.03
N11	ESI-	281.25	133.64 ± 4.79	3.58	16.99 ± 0.00	0.03
N12	ESI-	283.26	62.78 ± 4.22	6.72	18.01 ± 0.00	0.03

[a] ESI+: ESI positive mode; ESI-: ESI negative mode.

Table S3. Identified metabolites and their relative levels in the control, model and LR groups.

Class	Metabolites	HMDB	Model/Control [a]	LR/Control [b]	LR/Model [c]	Analytical Instrument [d]
Fatty acids	Docosahexaenoic acid (DHA)	HMDB0002183	↓***	↓	↑***	LC-MS (ESI-)
Fatty acids	Arachidonic acid	HMDB0001043	↓***	↓***	↑**	LC-MS (ESI-)
Fatty acids	Linoleic acid	HMDB0000673	↑***	↑***	↓***	LC-MS (ESI-)
Fatty acids	Stearidonic Acid	HMDB0006547	↑***	↑**	↓	LC-MS (ESI+)
Fatty acids	Paullinic acid	HMDB0035159	↑***	↑***	↓	LC-MS (ESI-)
Fatty acids	Thromboxane B2	HMDB0003252	↑***	↑***	↓	LC-MS (ESI-)
Fatty acids	9-HOTE	HMDB0010224	↑***	↑**	↓	LC-MS (ESI+)
Fatty acids	9(S)-HPODE	HMDB0006940	↑***	↑**	↓	LC-MS (ESI-)
Fatty acids	9,12,13-TriHOME	HMDB0004708	↑**	↑*	↓	LC-MS (ESI-)
Fatty acids	13-OxoODE	HMDB0004668	↑**	↑**	↓	LC-MS (ESI+)
Fatty acids	13-HODE	HMDB0004667	↑***	↑***	↓	LC-MS (ESI-)
Fatty acids	14,15-EpETrE	HMDB0004264	↑**	↑***	↓	LC-MS (ESI+)
Fatty acids	15(S)-HPETE	HMDB0004244	↑***	↑**	↓	LC-MS (ESI-)
Amino acids	L-Tyrosine	HMDB0000158	↓*	↓	↑	GC-MS
Amino acids	N-Lauroylglycine	HMDB0013272	↓***	↓**	↑	LC-MS (ESI-)

Amino acids	Phosphopantothenic acid	HMDB0001016	↑***	↑***	↓***	LC-MS (ESI-)
Lipids	Glycerol 3-phosphate	HMDB0000126	↓**	↓	↑*	GC-MS
Lipids	LysoPE(0:0/16:0)	HMDB0011473	↑***	↑***	↓	LC-MS (ESI+)
Lipids	LysoPE(20:1(11Z)/0:0)	HMDB0011512	↑**	↑***	↓	LC-MS (ESI+)
Lipids	PE(20:1(11Z)/16:0)	HMDB0009253	↑***	↑***	↓	LC-MS (ESI-)
Lipids	4-oxo-Retinoic acid	HMDB0006285	↑***	↑***	↓	LC-MS (ESI-)
Lipids	Corticosterone	HMDB0001547	↑***	↑**	↓	LC-MS (ESI-)
Nucleosides	Adenosine monophosphate (AMP)	HMDB0000045	↓**	↓**	↑*	LC-MS (ESI-)
Nucleosides	Adenosine	HMDB0000050	↓*	↓*	↑	LC-MS (ESI+)
Nucleosides	Inosine	HMDB0000195	↓**	↓**	↑	LC-MS (ESI+)
Nucleosides	5'-Methylthioadenosine (MTA)	HMDB0001173	↓***	↓***	↑	LC-MS (ESI+)
Nucleosides	Arabinosylhypoxanthine	HMDB0003040	↓***	↓***	↑	LC-MS (ESI-)
Nucleosides	Dephospho-CoA	HMDB0001373	↓***	↓***	↑	LC-MS (ESI-)
Others	Succinic acid	HMDB0000254	↑	↑	↓*	GC-MS
Others	Choline	HMDB0000097	↑***	↑*	↓**	LC-MS (ESI+)
Others	Erythronic acid	HMDB0000613	↓*	↓***	↑	LC-MS (ESI+)
Others	3-Hydroxybutyric acid	HMDB0000357	↓***	↓**	↓	GC-MS
Others	N-Acetylmannosamine	HMDB0001129	↑***	↑**	↓	GC-MS
Others	7'-Carboxy-gamma-tocotrienol	HMDB0012851	↑***	↑**	↓	LC-MS (ESI-)

[a] Comparison of relative levels of metabolites in the model group and the control group.

[b] Comparison of relative levels of metabolites in the LR group and the control group.

[c] Comparison of relative levels of metabolites in the LR group and the model group.

[d] ESI+: ESI positive mode; ESI-: ESI negative mode.

The hypothesis test for the difference between two means was conducted through t-test. *: $p < 0.05$; **: $p < 0.01$; ***: $p < 0.001$.
